# The efficacy of high-flow nasal cannula (HFNC) versus non-invasive ventilation (NIV) in patients at high risk of extubation failure: a systematic review and meta-analysis

**DOI:** 10.1186/s40001-023-01076-9

**Published:** 2023-03-14

**Authors:** Qiaoying Wang, Yanchun Peng, Shurong Xu, Lingyu Lin, Liangwan Chen, Yanjuan Lin

**Affiliations:** 1 Department of Cardiovascular Surgery, Fujian Medical University Union Hospital, No. 29, Xinquan Road, Fuzhou, Fujian China; 2 School of Nursing, Fujian Medical University, No. 1, Xuefu North Road, Fuzhou, Fujian China; 3 Department of Nursing, Fujian Medical University Union Hospital, No. 29, Xinquan Road, Fuzhou, Fujian China

**Keywords:** High-flow nasal cannula, Extubation failure, High-risk patient, Meta-analysis

## Abstract

**Background:**

Studies suggest that high-flow nasal cannula (HFNC) and non-invasive ventilation (NIV) can prevent reintubation in critically ill patients with a low risk of extubation failure. However, the safety and effectiveness in patients at high risk of extubation failure are still debated. Therefore, we conducted a systematic review and meta-analysis to compare the efficacies of HFNC and NIV in high-risk patients.

**Methods:**

We searched eight databases (MEDLINE, Cochrane Library, EMBASE, CINAHL Complete, Web of Science, China National Knowledge Infrastructure, Wan-Fang Database, and Chinese Biological Medical Database) with reintubation as a primary outcome measure. The secondary outcomes included mortality, intensive care unit (ICU) length of stay (LOS), incidence of adverse events, and respiratory function indices. Statistical data analysis was performed using RevMan software.

**Results:**

Thirteen randomized clinical trials (RCTs) with 1457 patients were included. The HFNC and NIV groups showed no differences in reintubation (RR 1.10, 95% CI 0.87–1.40, *I*^*2*^ = 0%, *P* = 0.42), mortality (RR 1.09, 95% CI 0.82–1.46, *I*^*2*^ = 0%, *P* = 0.54), and respiratory function indices (partial pressure of carbon dioxide [PaCO_2_]: MD − 1.31, 95% CI − 2.76–0.13,* I*^*2*^ = 81%, *P* = 0.07; oxygenation index [P/F]: MD − 2.18, 95% CI − 8.49–4.13, *I*^*2*^ = 57%, *P* = 0.50; respiratory rate [Rr]: MD − 0.50, 95% CI − 1.88–0.88,* I*^*2*^ = 80%, *P* = 0.47). However, HFNC reduced adverse events (abdominal distension: RR 0.09, 95% CI 0.04–0.24, *I*^*2*^ = 0%, *P* < 0.01; aspiration: RR 0.30, 95% CI 0.09–1.07, *I*^*2*^ = 0%, *P* = 0.06; facial injury: RR 0.27, 95% CI 0.09–0.88, *I*^*2*^ = 0%, *P* = 0.03; delirium: RR 0.30, 95%CI 0.07–1.39, *I*^*2*^ = 0%, *P* = 0.12; pulmonary complications: RR 0.67, 95% CI 0.46–0.99, *I*^*2*^ = 0%, *P* = 0.05; intolerance: RR 0.22, 95% CI 0.08–0.57, *I*^*2*^ = 0%, *P* < 0.01) and may have shortened LOS (MD − 1.03, 95% CI − 1.86–− 0.20, *I*^*2*^ = 93%, *P* = 0.02). Subgroup analysis by language, extubation method, NIV parameter settings, and HFNC flow rate revealed higher heterogeneity in LOS, PaCO_2_, and Rr.

**Conclusions:**

In adult patients at a high risk of extubation failure, HFNC reduced the incidence of adverse events but did not affect reintubation and mortality. Consequently, whether or not HFNC can reduce LOS and improve respiratory function remains inconclusive.

**Supplementary Information:**

The online version contains supplementary material available at 10.1186/s40001-023-01076-9.

## Background

Due to the complexity and variability of conditions, patient life is sustained in Intensive Care Units (ICUs) using mechanical ventilation. Approximately 39.0%–72.0% of ICU patients require invasive mechanical ventilation, and reintubation after mechanical ventilation is considered a potentially adverse event [1–4]. Previous studies have shown that 10%–20% of extubated patients require reintubation, and that 20–30% have a high risk of extubation failure [4–6]. Reintubation is associated with a longer hospital stays, poor prognosis, higher healthcare costs, and more complications [6–8].

To reduce reintubation, a prophylactic high-flow nasal cannula (HFNC) and non-invasive mechanical ventilation (NIV) after extubation are used to improve oxygenation in patients at high-risk of extubation failure. HFNC delivers a heated, humidified, and adjustable air–oxygen mixture through a large-caliber nasal cannula. NIV assists the patient’s breathing by applying different levels of positive pressure to the airway through an oral or nasal mask, without endotracheal intubation or laryngeal mask airway insertion. Nava et al. [9] and Ferrer et al. [10] demonstrated that NIV helped to reduce respiratory failure, reintubation, and mortality after extubation in high-risk patients. However, NIV can cause many side effects, such as lung damage, gastric distension, mask discomfort, and even claustrophobia, conditions that are difficult for patients endure [11, 12]. Numerous studies have shown that HFNC is not inferior to NIV in preventing the reintubation of high-risk patients. In addition, HFNC was better tolerated, more comfortable, and resulted in fewer adverse events than NIV [13–15]. The international clinical practice guidelines indicate that when compared with HFNC, NIV is more effective in preventing reintubation. However, HFNC does not lead to adverse events among high-risk patients [16].

Therefore, we conducted a systematic review and meta-analysis based on randomized-controlled trials (RCTs) to evaluate the role of HFNC in preventing reintubation, mortality, and adverse events as well as improving respiratory function, and shortening ICU length of stay (LOS).

## Methods

The Preferred Reporting Items for Systematic Reviews and Meta-Analyses (PRISMA) [17] checklist was used to report the systematic review and meta-analysis (Materials Additional file 1), and we registered the study with PROSPERO (CRD42022311969).

### Search strategy

Two investigators (W-Q and P-Y) independently searched the MEDLINE, Cochrane Library, EMBASE, CINAHL Complete, Web of Science, China National Knowledge Infrastructure, Wan-Fang database, and Chinese Biological Medical Database for relevant studies. The retrieval dates ranged from the establishment of the database until February 28, 2022. The retrieval strategy combined non-invasive ventilation (‘niv’ or ‘nippv’ or ‘noninvasive ventilation’ or ‘non invasive positive pressure ventilation’ or ‘noninvasive positive pressure ventilation’ or ‘non invasive ventilation’) with high-flow nasal cannula(‘hhfnc’ or ‘hhfn’ or ‘hfnc’ or ‘hfnct’ or ‘high flow nasal cannula’ or ‘high flow nasal cannula oxygen therapy’ or ‘nasal high flow oxygen therapy’ or ‘high flow nasal cannulae’ or ‘humidified high flow nasal cannula therapy’). See Material Additional file 2, for example, Medline search.

### Study selection

The following inclusion criteria were used: (1) patients at high risk of extubation failure who fulfilled at least one of the following [9, 10, 13, 16, 18–20]: age older than 65 years, underlying cardiac or respiratory disease, mechanical ventilation time > 7 days, airway patency problems including ineffective cough or excessive tracheobronchial secretion, an Acute Physiology and Chronic Health Evaluation II (APACHE II) score > 12 on extubation day, body mass index (BMI) > 30 kg/m^2^, and 2 or more comorbidities; (2) HFNC and NIV were used as interventions; (3) outcomes including, but not limited to, reintubation, mortality, LOS, the incidence of adverse events (abdominal distension, aspiration, facial injury, delirium, pulmonary adverse events, and intolerance), and respiratory function indices (partial pressure of carbon dioxide [PaCO_2_], oxygenation index [PaO_2_/FiO_2_, P/F], respiratory rate [Rr]); (4) RCTs as the study type; (5) and studies either in Chinese or English language.

The exclusion criteria included insufficient information, incomplete data; age < 18 years; repeated publications; and high-risk bias literature (Grade C).

### Data extraction and quality assessment

Data were independently extracted by two researchers (W-Q and X-S), and any discrepancies were resolved by a third researcher (P-Y). The following data were extracted from the selected studies: first author, year of publication, country, disease type, sample sizes, gender, age, intervention measures, reintubation, mortality, LOS, adverse events, and respiratory function-related indices. The quality of each RCT that satisfied the criteria was examined using the Cochrane bias risk assessment tool (Cochrane 5.1.0) [21]. The bias risk was classified into three categories: Grade A, items are all low risk of bias; Grade C, items are all high risk of bias; and Grade B, items are not all high or low risk of bias. In addition, two researchers independently assessed the credibility of the pooled results using GRADE guidelines. The results of the meta-analysis were assessed using five downgrades and three upgrades, and the credibility of the results was classified as high, moderate, low, or very low.

### Outcome measures

The primary outcome was reintubation. The secondary outcomes were mortality, LOS, the incidence of adverse events (abdominal distension, aspiration, facial injury, delirium, pulmonary adverse events, and intolerance), PaCO_2_, P/F, and Rr.

### Statistical analysis

RevMan5.4.1 software was used to create forest maps and merge data. Dichotomous data, such as reintubation, mortality, and incidence of adverse events, are expressed as a risk ratios (RR) and 95% confidence intervals (CI). Continuous data, such as LOS and respiratory function-related indices, are expressed as a mean difference (MD) and 95% CI. A *P* < 0.05 was considered statistically significant.

We used the Cochran’s *Q* test and* I*^*2*^ test statistics to test the heterogeneity of the studies. A *P* > 0.05 and* I*^*2*^ < 50%, indicated low heterogeneity, and a fixed-effects model (FD) was used. A *P* < 0.05 or I^2^ > 50% indicated high, and a random-effects model (RD) was used. Subgroup analysis of each outcome was carried out by language (English versus Chinese), extraction method (conventional versus non-conventional), NIV parameter settings (fixed expiratory positive airway pressure [EPAP] and inspiratory positive airway pressure [IPAP] versus non-fixed), and HFNC flow rate (fixed versus non-fixed) for comparison between HFNC and NIV. At the same time, we performed sensitivity analysis by removing one study at a time to ensure the stability of the results. Funnel plots were used applied to detect publication bias in more than ten trials.

## Results

### Study selection and characteristics

**Fig. 1:**
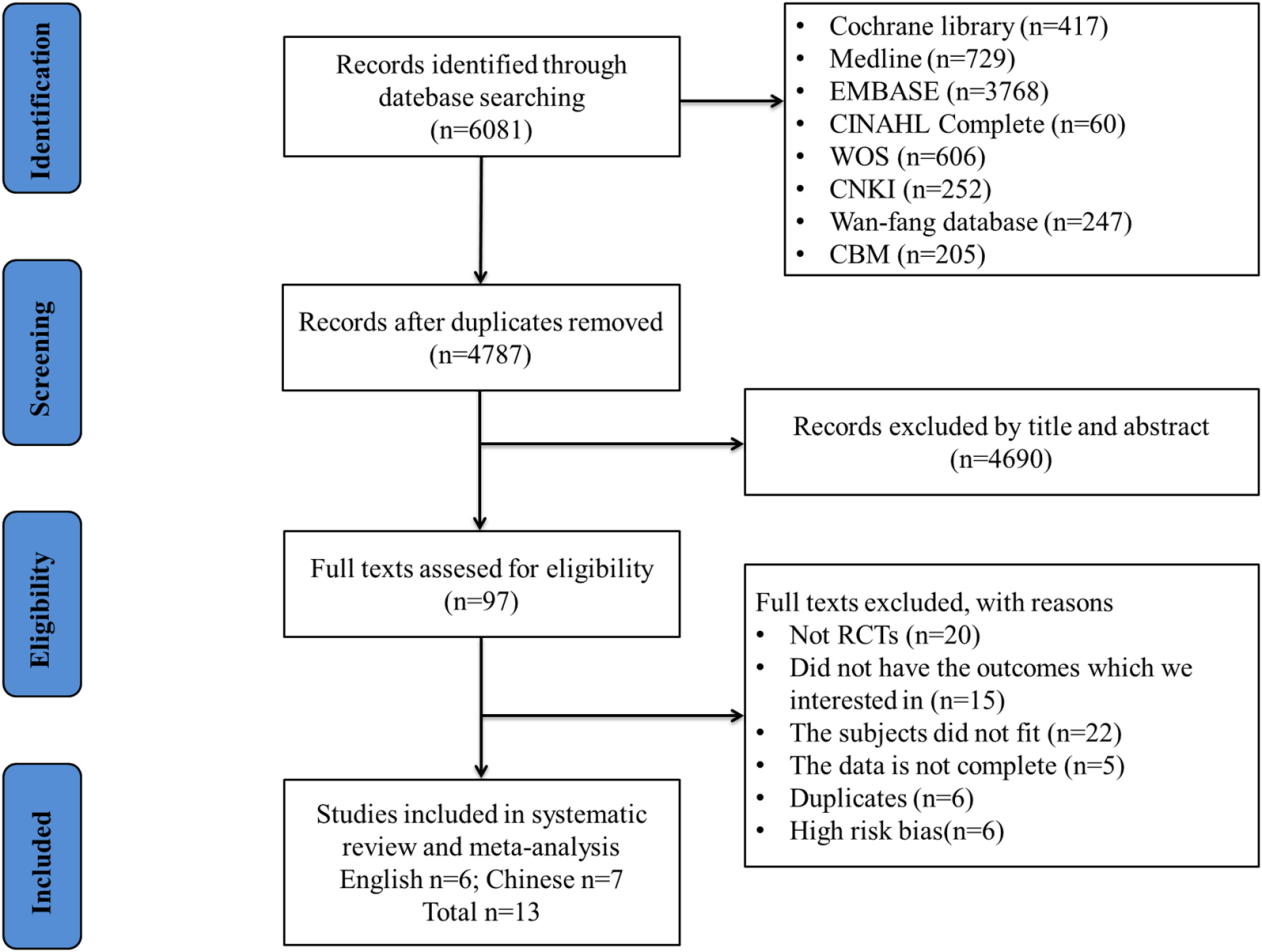
The PRISMA flow diagram of selected studies. WOS: Web of Science, CNKI: China National Knowledge Infrastructure, CBM: Chinese Biological Medical Database

After removing the duplicate articles, a total of 4787 of 6282 articles were available for analysis. Of the total, 97 articles were selected for full-text review after reading the titles and abstracts of the publications, and finally, 13 RCTs [13, 15, 22–32], including 1457 patients, were eventually included. The screening process is shown in Fig. 1. Of the 13 RCTs included, 6 were published in English [13, 15, 27, 30–32] and 7 in Chinese [22–26, 28, 29]. The included study objects included chronic obstructive pulmonary disease (COPD) [15, 23–28, 32], APACHEII > 12 [30], elderly patients (> 65) with sepsis [31], patients at risk of extubation failure [13], acute pancreatitis with respiratory distress syndrome [29], and hypoxemia after cardiac surgery [22]. The mean age in 9 of the RCTs was > 65 years [15, 23, 24, 26, 27, 30–32], and 5 of the RCTs < 65 years [13, 22, 25, 28, 29]. One RCT was identified as Grade A, and 12 RCTs were identified as Grade B (Table 1). Nine of the RCTs [13, 22–24, 27–31] were on routine standard extubation, and four [15, 25, 26, 32] were on unconventional extubation following the pulmonary infection control window (PIC).

**Table 1:** Basic characteristics of the study were included

Study	Year	Region	Disease	Sample(n)		Sex (male/female)		Age ($\overline{x }$±s, years)		Intervention		Grade (risk of bias)	Included outcomes
				HFNC	NIV	HFNC	NIV	HFNC	NIV	HFNC	NIV		
Hernández et al	2016	France	High-risk patients	290	314	186/104	202/112	64.6 ± 15.4	64.4 ± 15.8	37℃, 10L/min, 5L/min was increased until the patient felt unwell	IPAP and EPAP were adjusted to target a Rr of 25/ min and adequate gas exchange	A	(1)(2)(3)(4)(5)(6)
Hu et al	2018	China	AECOPD	60	60	34/26	30/30	NA	NA	37℃, 40L/min, keep SpO_2_ > 92%	IPAP 10–12 cm H_2_O, EPAP 4–6 cmH_2_O	B	(1)(4)(5)(7)
Jiang et al	2019	China	AECOPD	18	17	13/5	11/6	75.0 ± 5.1	71.0 ± 6.5	50L/min, keep SpO_2_ > 92%	5–8cmH_2_O EPAP, keep SpO_2_ > 92%	B	(1)(3)(4)(5)(7)
Jing et al	2019	China	COPD	22	20	NA	NA	77.4 ± 6.8	73.9 ± 6.9	37℃, keep SpO_2_ 88%–92%	IPAP 10–12 cmH_2_O, EPAP 4–5cmH2O	A	(1)(2)(3)(4)(5)(6)(7)
Liu et al	2019	China	COPD	44	43	24/20	22/21	64.1 ± 10.5	65.1 ± 9.7	20–40 L/min	IPAP 4 cmH_2_O, 5–10 min increased 2 cmH_2_O, keep 8–20cmH_2_O, EPAP 4–6cmH_2_O	B	(1)(2)(5)(7)
Shang et al	2021	China	APACHEII > 12	24	24	12/12	13/11	66.92 ± 4.58	67.7 ± 6.89	37℃, 40L/min, keep SpO_2_ > 90%	10–12cmH_2_O IPAP, 4–6cmH_2_O EPAP	B	(1)(2)(3)(4)(5)(7)
Tan et al	2020	China	COPD	44	42	27/17	24/19	68.4 ± 9.3	71.4 ± 7.8	37℃, 50L/min, keep SpO_2_ 88%–92%	8cmH_2_O IPAP, 4cmH_2_O EPAP	A	(1)(2)(3)(4)(5)(6)(7)
Tongyoo et al	2021	Thai-land	> 65	60	58	NA	NA	> 65	> 65	37℃, 30L/min, 10 min increased 5L/min until 50L/min	8cmH_2_O IPAP, 5cmH_2_O EPAP, IPAP increased 2cmH_2_O per 10 min	A	(1)
Wang et al	2021	China	SAP with ARDS	30	30	25/5	24/6	43 ± 7	41 ± 7	37℃, 40–50L/min, keep SpO_2_ > 95%	IPAP 5–12cmH_2_O, EPAP 0–5cmH_2_O	B	(1)(2)(3)(4)(5)(6)(7)
Xu et al	2021	China	COPD	50	50	32/18	30/20	69.2 ± 6.13	68.3 ± 5.22	37℃, FiO_2_ 30%–50%, 50 L/min, keep SpO_2_ 88%–92%	EPAP 4–5mmH_2_O, IPAP 10–12mmH_2_O, keep SpO_2_ 88%-92%	B	(1)(4)
Yang et al	2015	China	Post- cardiac surgery	20	20	14/6	13/2	53.8 ± 8.9	52.9 ± 7.8	37℃, 45 L/min	IPAP 10–12cmH_2_O, EPAP 4–6 cmH_2_O	B	(1)(2)(3)(4)(5)(6)(7)
Yu et al	2019	China	COPD	36	36	24/12	21/15	62.4 ± 10.1	63.5 ± 11.2	37℃, 30–60 L/min, FiO_2_ 30%–80%	IPAP 10–14 cmH_2_O, EPAP 4–6 cmH_2_O	B	(1)(2)(4)(5)(6)(7)
Zhang et al	2018	China	COPD	21	24	18/3	20/4	64.5 ± 5.3	66.1 ± 6.6	37℃, 40L/min, when SpO_2_ = 92%, change 30L/min	IPAP 5–15 cmH_2_O, EPAP 0–5cmH_2_O	B	(1)(2)(3)(4)(5)(6)

HFNC: high-flow nasal cannula oxygen therapy, NIV: non-invasive ventilation, AECOPD: acute exacerbation of chronic obstructive pulmonary disease, COPD: chronic obstructive pulmonary disease, SAP: severe acute pancreatitis, ARDS: acute respiratory distress syndrome, IPAP: inspiratory positive airway pressure, EPAP: expiratory positive airway pressure, Reintubation rates

(1) Reintubation rates

(2) Mortality rate

(3) ICU length of stay

(4) Incidence of adverse events

(5) Partial pressure of carbon dioxide

(6) Oxygenation index

(7) Respiratory rate

**Fig. 2:**
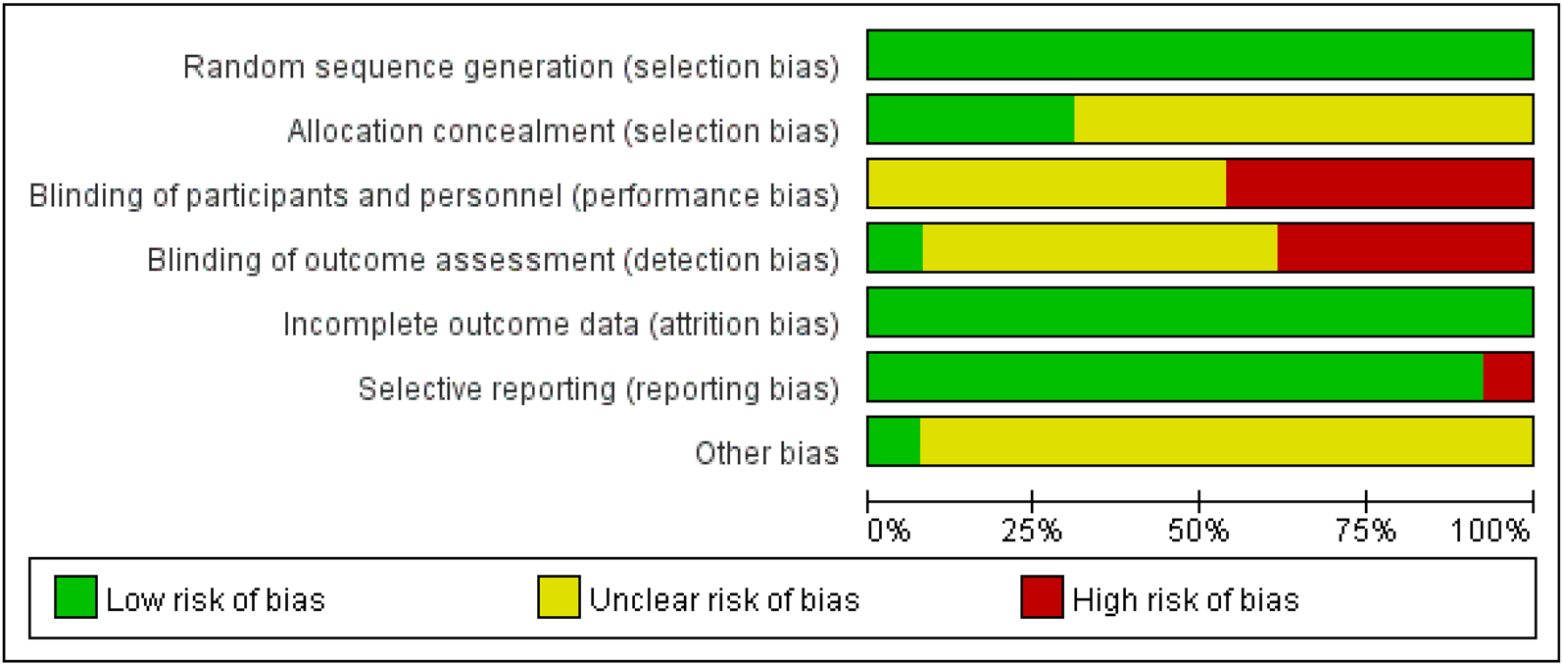
Risk of bias graph: each risk of bias item presented as percentages across studies

**Fig. 3:**
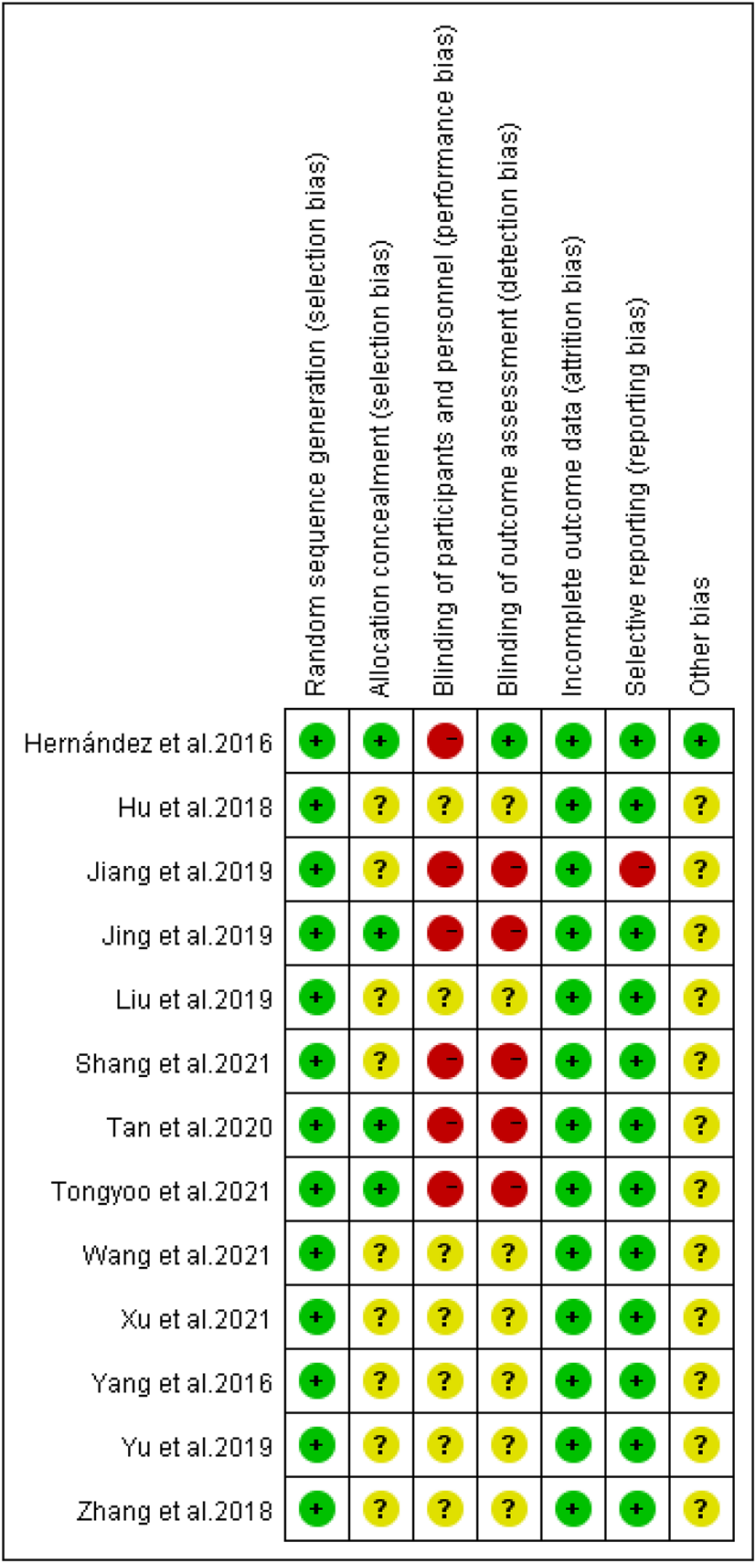
Risk of bias summary: judgements about each risk of bias item for each study. Green circles indicate a low risk of bias, yellow circles indicate an ambiguous risk of bias, and red circles indicate a high risk of bias

The 13 included RCTs were assessed for risk of bias using the Cochrane risk assessment tool. Of the 13 total RCTs, 5 used computer number generators, 8 used the random table to ensure adequate random sequence generation, 6 described allocation concealment, 1 adopted the single-blind method, 5 did not adopt the blind method, and 7 did not know whether to apply the blind method. All the literature results and data were reported thoroughly. One RCT reported research findings selectively, and other sources of bias in the remaining 12 RCTs were unclear. Figure 2 shows a summary of the risk of bias. Figure 3 shows the risk map of bias. Blinding participants, interveners, and outcome measures in studies are challenging, and therefore, we classified most studies as having a high risk of bias, because they had not been blinded.

### Efficacy of HFNC

#### Reintubation

**Fig. 4:**
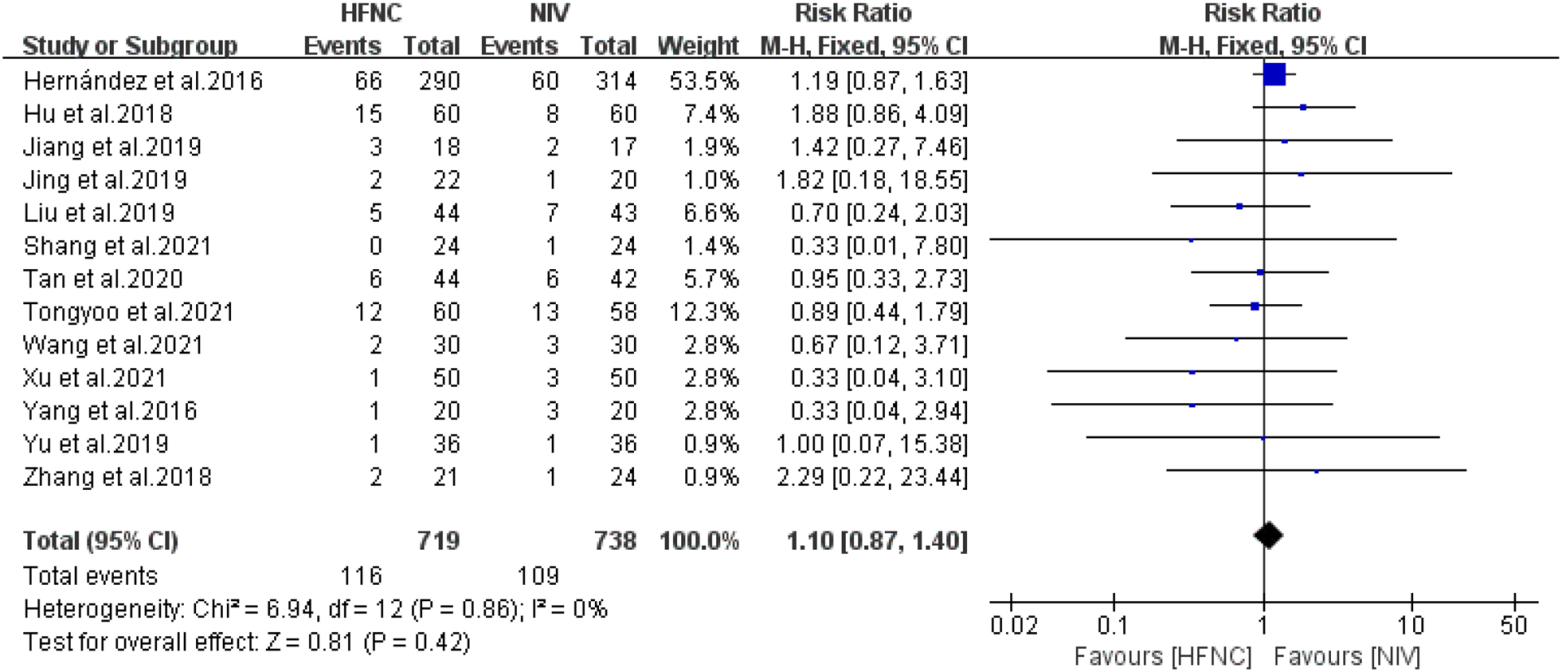
Forest plots for reintubation between high-flow nasal cannula and non-invasive ventilation

The effect of HFNC versus NIV on the reintubation rate of patients at high risk for extubation failure was described in the 13 included RCTs. We found low heterogeneity across the studies, and the FD was performed. The meta-analysis revealed no significant difference in reintubation rates between the HFNC and NIV groups (*n* = 1457, *I*^*2*^ = 0%, RR = 1.10, 95%CI = 0.87–1.40, *P* = 0.42) Fig. 4.

#### Mortality

**Fig. 5:**
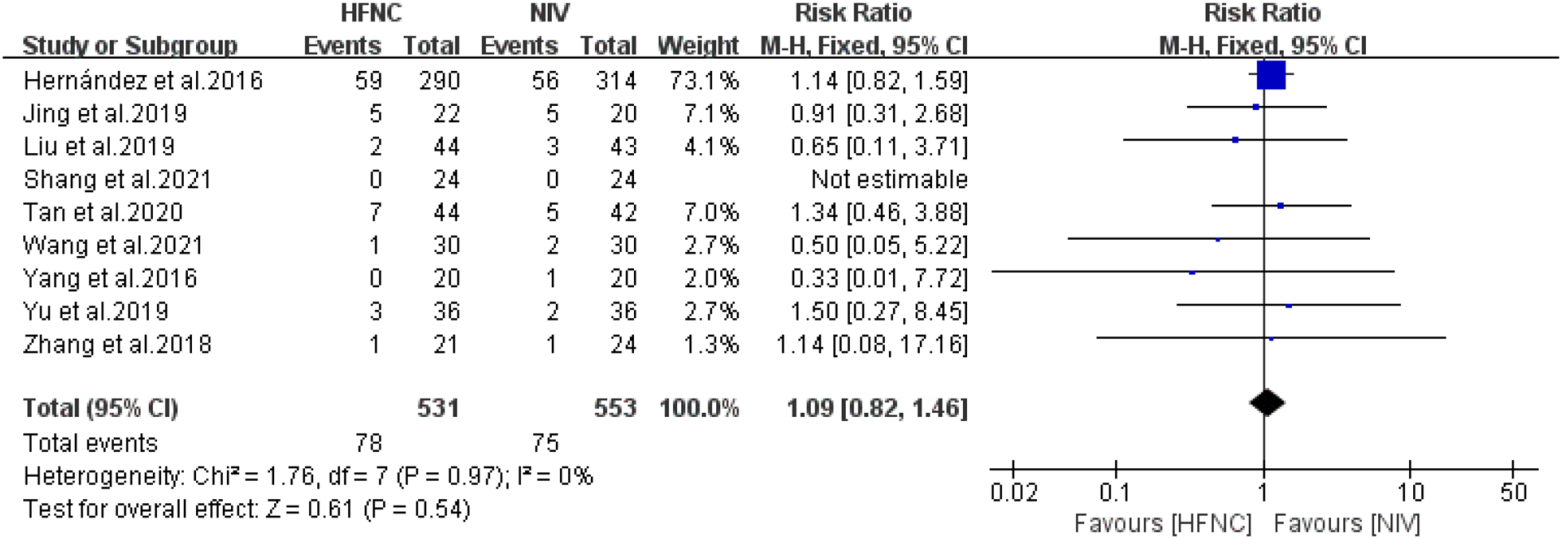
Forest plots for mortality between high-flow nasal cannula and non-invasive ventilation

Nine RCTs described the effect of HFNC versus NIV on the mortality of patients at high risk of extubation failure. Low heterogeneity was found across the studies, and FD was performed. The meta-analysis revealed no significant differences in mortality between the HFNC and NIV groups (*n* = 1084, *I*^*2*^ = 0%, RR = 1.09, 95%CI = 0.82–1.42, *P* = 0.54) Fig. 5.

#### ICU length of stay

**Fig. 6:**
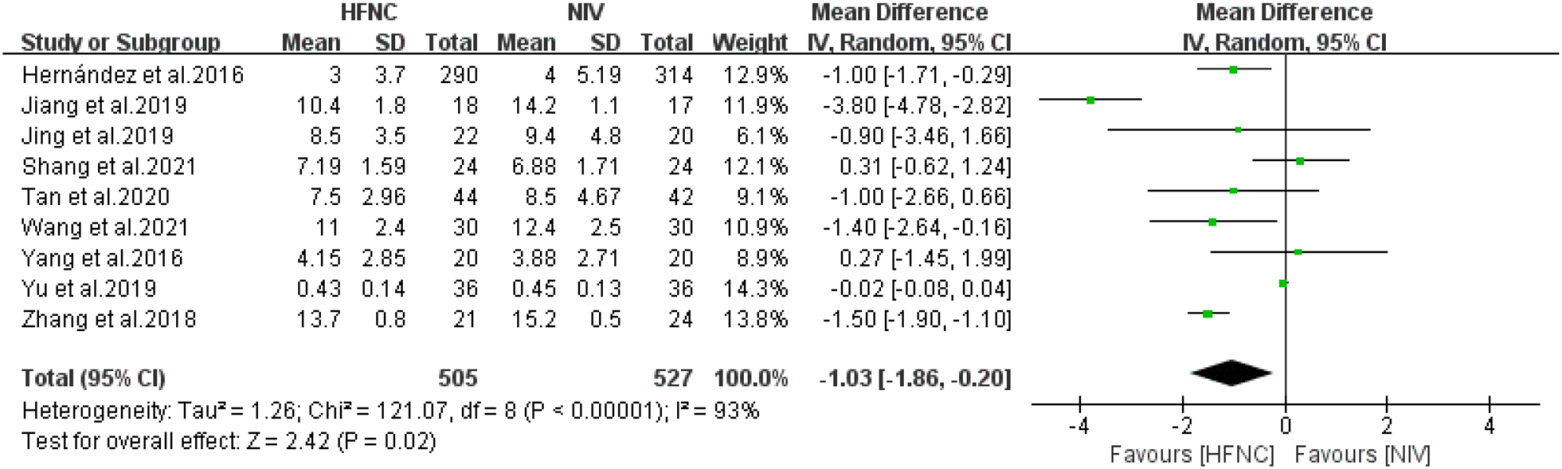
Length of ICU stay forest plot between high-flow nasal cannula and non-invasive ventilation

The effect of HFNC versus NIV on the LOS of patients at a high risk of extubation failure was described in 9 of the 13 included RCTs. As high heterogeneity was found across the studies, and RD was performed. As a result, we found that HFNC had a significant advantage over NIV (*n* = 1032, *I*^*2*^ = 93%, RR = − 1.03, 95%CI = − 1.86–0.20, *P* = 0.02) Fig. 6.

#### Incidence of adverse events

**Fig. 7:**
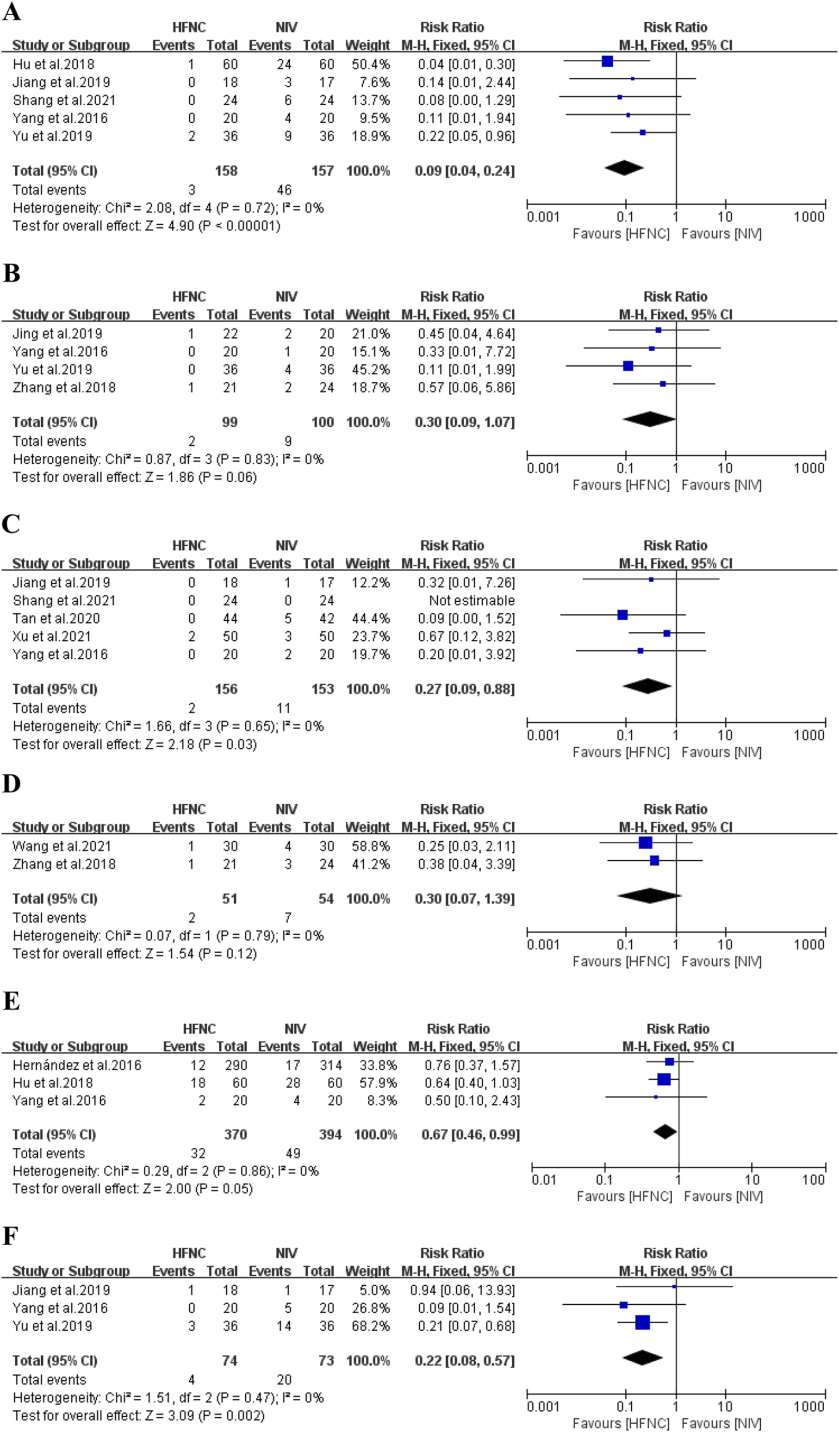
Forest plots for abdominal distension **A**, aspiration **B**, facial injury **C**, delirium **D**, pulmonary complications **E**, and intolerance **F**

In 11 of the 13 included RCTs, the effect of HFNC versus NIV on the incidence of adverse events, including abdominal distension, aspiration, facial injury, delirium, pulmonary complications, and intolerance in patients at a high risk of extubation failure, was described. We found low heterogeneity across the studies, and FD was performed. A meta-analysis revealed that HFNC presented clear advantages over NIV in abdominal distension, facial injury, pulmonary complications, and intolerance (abdominal distension: *n* = 315, *I*^*2*^ = 0%, RR 0.09, 95% CI 0.04–0.24,* P* < 0.01; facial injury: *n* = 309, *I*^2^ = 0%, RR = 0.27, 95% CI 0.09–0.88, *P* = 0.03; pulmonary complications: n = 764, *I*^*2*^ = 0%, RR 0.67, 95% CI 0.46–0.09, *P* = 0.05; intolerance: *n* = 147, *I*^*2*^ = 0%, RR 0.22, 95% CI 0.08–0.57, *P* < 0.01). No significant differences in aspiration or delirium were found between the HFNC and NIV groups (aspiration: *n* = 199, *I*^*2*^ = 0%, RR 0.30, 95% CI 0.09–1.07, *P* = 0.06; delirium: *n* = 105, *I*^*2*^ = 0%, RR 0.30, 95% CI 0.07–1.39, *P* = 0.12) Fig. 7.

#### Respiratory function-related indices

**Fig. 8:**
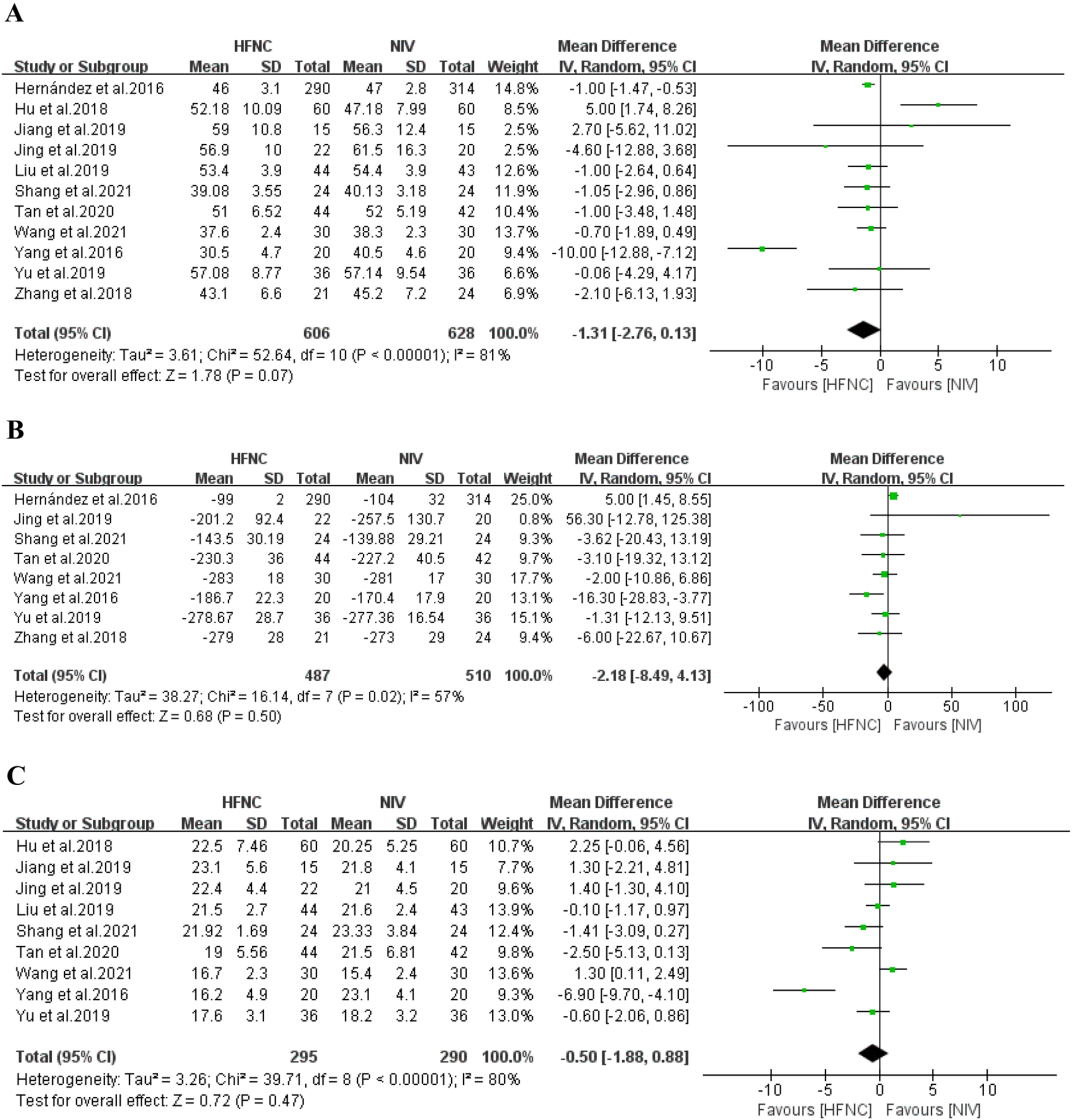
Forest plots for partial pressure of carbon dioxide **A**, oxygenation index **B**, and respiratory rate **C**

In 11 of the 13 included RCTs, the effect of HFNC versus NIV on the PaCO_2_, P/F, and Rr of patients at high risk of extubation failure was described. Of the total, 11 RCTs described PaCO_2_, 8 described P/F, and 9 described Rr. High heterogeneity was found in all outcomes, and RD was performed. The results showed that HFNC provided several advantages over NIV (PaCO_2_: *n* = 1234, *I*^*2*^ = 81%, MD − 1.31, 95% CI − 2.76–0.13, *P* = 0.07; P/F: *n* = 997, *I*^*2*^ = 57%, MD − 2.18, 95% CI − 8.49–4.13, *P* = 0.50; Rr: *n* = 585, *I*^*2*^ = 80%, MD − 0.50, 95% CI − 1.88–0.88, *P* = 0.47) Fig. 8.

### Subgroup analysis

Subgroup analyses of reintubation, mortality, LOS, adverse events, and respiratory function-related indices by language, extraction method, NIV parameter settings, and HFNC flow rate to compare HFNC versus NIV. Our results showed no significant differences or reductions in heterogeneity regarding reintubation, mortality, abdominal distension, aspiration, and Rr. In LOS, with exception of the Chinese-language group (*n* = 252, *I*^*2*^ = 96%, RR = − 1.32, 95%CI = − 2.56–− 0.09, *P* = 0.04), we did find statistically significant differences in the other subgroup analyses (English language: *n* = 780, *I*^*2*^ = 41%, RR = − 0.55, 95%CI = − 1.34–0.24, *P* = 0.17; conventional extubation standards: *n* = 911, *I*^*2*^ = 91%, RR = − 0.62, 95%CI = − 1.37–0.63, *P* = 0.11; unconventional extubation standards: *n* = 121, *I*^*2*^ = 88%, RR = − 2.48, 95%CI = − 5.22–0.24, *P* = 0.08; unfixed NIV setting: *n* = 128, *I*^*2*^ = 94%, RR = − 1.65, 95%CI = − 3.49—0.18, *P* = 0.08; fixed NIV setting: *n* = 904, *I*^*2*^ = 64%, RR = − 0.59, 95%CI = − 1.25–− 0.06, *P* = 0.08; unfixed HFNC flow rate: *n* = 225, *I*^*2*^ = 90%, RR = − 1.15, 95%CI = − 2.99–0.70, *P* = 0.08; fixed HFNC flow rates: *n* = 807, *I*^*2*^ = 95%, RR = − 0.85, 95%CI = − 1.85–− 0.15, *P* = 0.10). The P/F in the subgroup analysis of language revealed no significant heterogeneity in the pooled results (English language: *n* = 780, *I*^*2*^ = 25%, RR = 2.91, 95%CI = − 4.48—10.29, *P* = 0.44; Chinese-language: *n* = 217, *I*^*2*^ = 25%, RR = − 1.18, 95%CI = − 12.27–0.47, *P* = 0.11). However, significant changes in the pooled results for PaCO_2_ were found in the English-language group, fixed NIV parameter settings, and fixed HFNC flow rate (English language: *n* = 780, *I*^*2*^ = 0%, RR = − 1.01, 95%CI = − 1.46–− 0.56, *P* < 0.01; fixed NIV setting: *n* = 991, *I*^*2*^ = 84%, RR = − 2.19, 95%CI = − 3.89–− 0.48, *P* = 0.01; fixed HFNC flow rates: *n* = 807, *I*^*2*^ = 0%, RR = − 1.00, 95%CI = − 1.46–− 0.54, *P* < 0.01). See Additional file 3 for details.

### Sensitivity analysis

Sensitivity analyses were performed by removing one study each for reintubation, mortality, LOS, adverse events, and respiratory function-related indices. Reintubation, mortality, LOS, and adverse events showed no significant changes in the pooled results or heterogeneity. However, heterogeneity was significantly reduced after excluding the study by Yang et al. for respiratory function-related indices (PaCO_2_: *I*^*2*^ = 40%, *P* = 0.14; P/F: *I*^*2*^ = 21%, *P* = 0.60; Rr: *I*^*2*^ = 57%, *P* = 0.80). See Table 2 for details.

**Table 2:** Sensitivity analysis on effects of HFNC on respiratory function indices

Outcome	Before sensitivity analysis			Remove study	After sensitivity analysis		
	Effect estimate	*P*	*I*^2^ (%)		Effect estimate	*P*	*I*^2^ (%)
PaCO_2_	− 1.31(− 2.76, 0.13)	0.07	81	Yang et al. 2016	− 0.63(− 1.46, -0.20)	0.14	40
PaO_2_/ FiO_2_	− 2.18(− 8.49, 4.13)	0.50	57	Yang et al. 2016	1.21(− 3.29, 5.71)	0.60	21
RR	− 0.50(− 1.88, 0.88)	0.47	80	Yang et al. 2016	− 0.13(− 0.85, 1.11)	0.80	57

Effect estimate: risk ratio (95% confidence interval)

PaCO_2_: partial pressure of carbon dioxide, P/F: oxygenation index, Rr: respiratory rate

### Assessment of publication bias

**Fig. 9:**
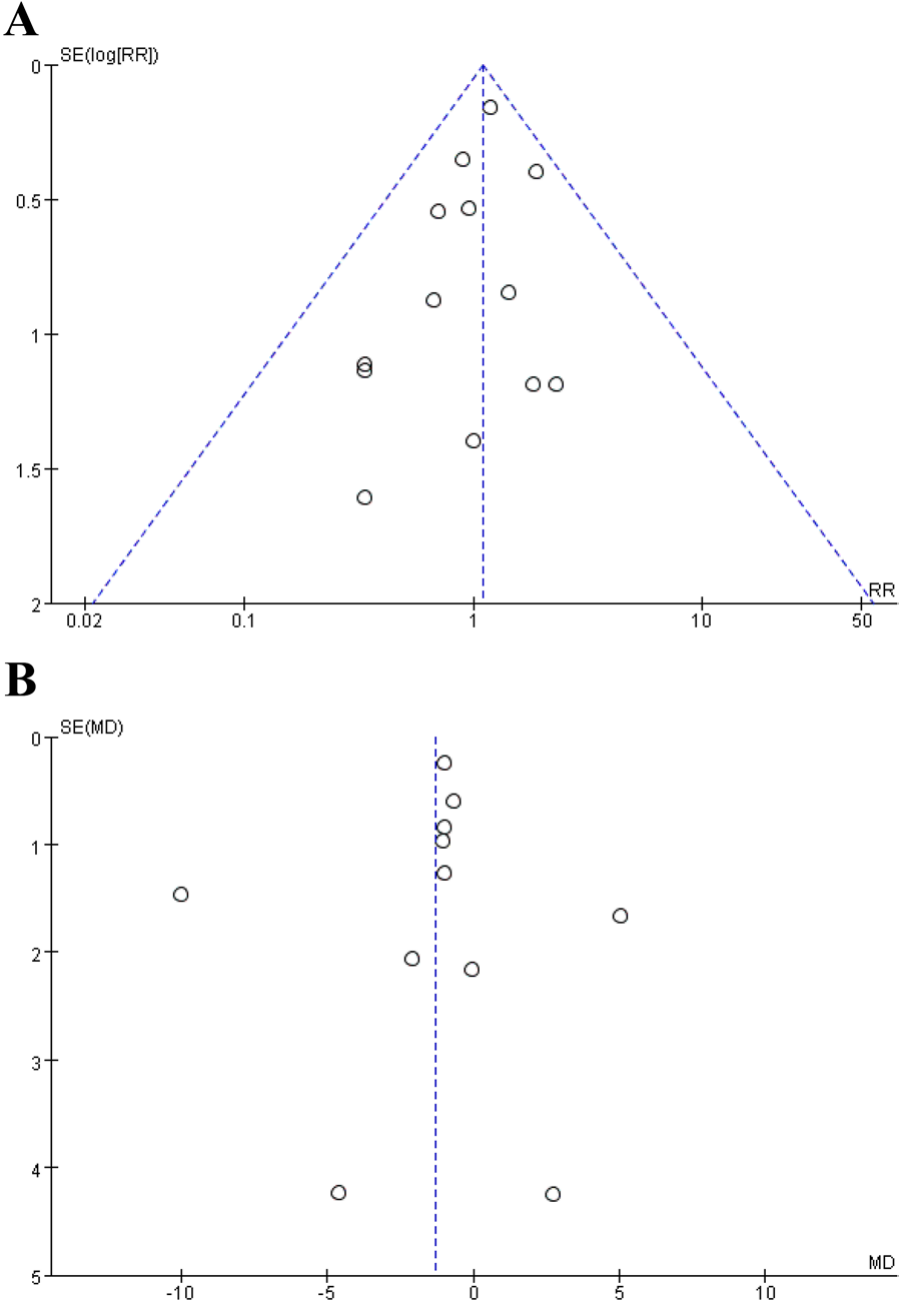
Funnel figure Reintubation **A** and partial pressure of carbon dioxide **B**

A funnel plot analysis was performed for meta-analyses of more than ten studies to assess publication bias. The funnel plots of the reintubation were symmetrical, suggesting a slight publication bias. The funnel plot for PaCO_2_ showed asymmetry, indicating a risk of publication bias Fig. 9.

### Certainty assessment

The certainty of the comprehensive assessment was evaluated using GRADE guidelines (Table 3). Heterogeneity and the risk of bias were the key factors that decreased the certainty of the evidence for most outcomes. Reintubation and facial injuries exhibited high certainty, abdominal distension, and pulmonary complications exhibited moderate certainty, and LOS exhibited low certainty.

**Table 3:** Summary of findings based on the GRADE guidelines for HFNC

Outcomes	Number of studies	Risk of bias	Inconsistency	Indirectness	Imprecision	Publication bias	Relative risk	Absolute effect	GRADE
Reintubation	13	No	No	No	No	Undetected	1.10 (0.87–1.40)	15/1000 (− 19–59)	⨁⨁⨁⨁ High
Mortality	9	No	No	No	Serious	Undetected	1.09 (0.82–1.46)	12/1000 (− 24–62)	⨁⨁⨁◯ Moderate
ICU stay	9	No	Serious^a^	No	Serious	Undetected	–	− 1.03 (− 1.86–− 0.2)	⨁⨁◯◯ Low
Abdominal distension	5	Serious^b^	No	No	No	Undetected	0.09 (0.04–0.24)	− 267/1000 (− 281–− 223)	⨁⨁⨁◯ Moderate
Aspiration	4	Serious^b^	No	No	No	Undetected	0.30 (0.09–1.07)	− 63/1000 (− 82–6)	⨁⨁⨁◯ Moderate
Facial injury	5	No	No	No	No	Undetected	0.27 (0.09–0.88)	− 52/1000 (− 65–− 9)	⨁⨁⨁⨁ High
Intolerance	3	Serious^b^	No	No	No	Strongly suspected^c^	0.22 (0.08–0.57)	− 214/1000 (− 252–− 118)	⨁⨁◯◯ Low
Pulmonary complications	3	No	No	No	No	Strongly suspected^c^	0.67 (0.46–0.99)	− 41/1000 (− 67–− 1)	⨁⨁⨁◯ Moderate
Delirium	2	Serious^d^	No	No	No	Strongly suspected^c^	0.30 (0.07–1.39)	− 91/1000 (− 121–51)	⨁⨁◯◯ Low
Partial pressure of carbon dioxide	11	No	Serious^e^	No	No	Strongly suspected^f^	–	− 1.31 (− 2.76–0.13)	⨁⨁◯◯ Low
Oxygenation index	8	No	No	No	Serious	Undetected	–	− 2.18 (− 8.49–4.13)	⨁⨁⨁◯ Moderate
Respiratory rate	9	No	Serious^a^	No	Serious	Undetected	–	− 0.5 (− 1.88–0.88)	⨁⨁◯◯ Low

^a^The level of heterogeneity was high and was not explained completely

^b^Many biased items are uncertain

^c^Number of included studies was too small

^d^Double blinding was not conducted

^e^The level of heterogeneity was more than 25%

^f^Funnel plots show publication bias

## Discussion

HFNC is a relatively new respiratory support technology that consisting of an air/oxygen blender, an actively heated humidifier, and a nasal cannula delivering up to 60 L/min of heated, humidified oxygen [33]. HFNC not only washes out the anatomical dead space, delivers positive end expiratory pressure (PEEP), maintains constant inspired oxygen concentration (FiO_2_), and provides sufficient humidification, but also it offers exceptional comfort and good applicability in the clinic [33–35]. Furthermore, it has been shown to be superior to traditional oxygen therapy. However, whether HFNC is preferable to NIV remains unclear in some patients, notably those with a high risk of extubation failure. Consequently, we conducted a systematic review and meta-analysis of the efficacy of HFNC therapy in patients at high risk of extubation failure. The findings demonstrated that the reintubation, mortality, and improvement in respiratory function in high-risk patients using HFNC as a preventive intervention were not inferior to those of NIV when the same parameters were compared. Moreover, fewer adverse effects, such as facial injuries and abdominal distension, were observed in patients using HFNC.

We analyzed the data from a total of 13 RCTs and found that HFNC was not inferior to NIV in preventing reintubation in patients at a high risk of extubation failure. This result was consistent with the conclusions of Hernández et al. [13] and Zhou et al. [36] but inconsistent with the recommendations of the European Respiratory Society guidelines published by Oczkowski et al. [16]. A possible reasons for this inconsistency could be the differing inclusion criteria for the interventions. The RCTs in our study excluded combined HFNC and NIV interventions; instead, we included only RCTs incorporating simple HFNC or simple NIV. Therefore, we excluded the publications by Thille et al. [37] who included large-sample multicentre RCT of up to 631 patients with a high risk of extubation failure, accounting for a significant weight in the meta-analysis. As a result, our findings are at odds with the recommendations. For mortality, our results showed no difference between the two groups, which is consistent with the studies by Oczkowski et al. [16] and Chang et al. [38].

Although consistent with the findings of Oczkowski et al. [16] and Hernández et al. [13], we found substantial heterogeneity and low certainty that HFNC would shorten the LOS. In our study, when subgroup analysis was carried out according to language, extraction method, NIV parameter settings, and HFNC flow rate, we discovered that with exception of the Chinese-language group, HFNC still demonstrated an advantage NIV. The English-language group and other subgroup analyses showed no statistical differences between HFNC and NIV. This finding may be related to a higher risk of literature bias in the Chinese-language group. Therefore, further research is necessary to determine whether HFNC therapy has a beneficial effect on the LOS.

Regarding adverse events, we discovered that HFNC reduced the incidence of abdominal distension, facial injury, pulmonary problems, and intolerance. Delirium and the aspiration were not significantly different between the HFNC and NIV groups; however, the HFNC group displayed an optimization trend. This result is in agreement with the findings of Hernández et al. [13] and Stéphan et al. [39].

Moreover, HFNC had the same effect on respiratory function indices as NIV, and improved PaCO_2_, P/F, and RR, which is consistent with the findings of Hernández et al. [13] and Jing et al. [27]. However, the results of those three indices revealed high heterogeneity. We found heterogeneity of P/F associated with language using subgroup analysis. The results of the Chinese-language group revealed no statistically significant differences between the two interventions, whereas those of the English-language group demonstrated that NIV was superior to HFNC. The reason for this discrepancy might be that the period of HFNC use in the Chinese-language articles was longer than that in the English-language articles. We also found that with respect to PaCO_2_, HFNC outperformed NIV when the HFNC flow rate was adjusted to the patient's condition; however, when the NIV parameters were adjusted, the two interventions were equivalent. Therefore, the personalized settings for the HFNC flow rate appeared to have a more significant effect on reducing CO_2_ retention than those of the NIV parameters. In addition, we carried out a sensitivity analysis, and found that the heterogeneity was dramatically reduced, and the pooled effect did not change significantly when the study by Yang et al. [22] was removed. We hypothesize that this finding was related to the study population, which included patients undergoing cardiac surgery, whereas other studies included patients with respiratory diseases. In patients with cardiac disease, increased flow in HFNC produces a degree of PEEP that causes a reduction in inferior vena cava collapse, subsequently reducing cardiac preload [40–42]. However, HFNC mainly decreases CO_2_ rebreathing by washing out the anatomical dead space and providing a steady oxygen concentration, which has an effective respiratory management effect in patients with respiratory diseases, especially COPD [43, 44]. Therefore, we believe that the heterogeneity was probably due to the different mechanisms of action between patients.

To ensure the certainty of the evidence, we used the GRADE system to rate each outcome. We also performed subgroup analyses due to the inclusion of English-language and Chinese-language articles, conventional extubation (satisfying extubation criteria), and unconventional extubation (intentional extubation in the presence of PIC). However, we did not have uniform inclusion criteria for the flow and temperature of HFNC and the inspiratory and expiratory air pressures of NIV. It is worth mentioning that although we performed subgroup analyses to determine whether the NIV parameters or HFNC flow rate changed, we could not perform more accurate subgroup analyses (e.g., comparing both NIV parameter settings and HFNC flow rate change, no change in both NIV parameter settings and HFNC flow rate, change in NIV setting parameters, and change in HFNC flow rate), because we included only 13 publications. Although our subgroup analysis confirmed that the NIV parameter settings and HFNC flow rate did not affect most study outcomes, it is undeniable that this does not completely eliminate the effect of such confounding factors. Therefore, future studies will require more refined settings for NIV parameters and HFNC flow rates. In addition, we only compared how HFNC alone and NIV alone affected high-risk patients of extubation failure with no discussion on whether NIV combined with HFNC is preferable to either one alone. According to Thille et al. [6, 45], HFNC combined with NIV significantly outperformed HFNC alone in reducing reintubation in high-risk patients with extubation failure. Therefore, future studies should focus on the effect of HFNC combined with NIV on patient comfort, durability, and prevention. Finally, the large-sample and multicentre RCT by Hernández et al. [13] holds sizable weight in our meta-analysis, which results in partial reliance on their findings and certain restrictions on our findings.

Currently, it is unclear whether HFNC and NIV are equally effective in preventing reintubation and how to define and diagnose high-risk patients with extubation failure. Consequently, more reliable clinical data are required to validate this finding.

## Conclusions

Among adult patients at high risk of extubation failure, HFNC is not inferior to NIV in preventing reintubation, mortality, and respiratory failure, and may shorten LOS, reduce adverse reactions, and increase patient comfort. However, heterogeneity of PaCO_2_, Rr, and LOS was evident in our results. Thus, a high-quality RCT is required to confirm whether HFNC therapy can provide an advantage in LOS and respiratory function in such patients.

## Supplementary Information

**Additional file 1. **PRISMA checklist.

**Additional file 2. **Medline search.

**Additional file 3: Figure S1.** Subgroup analysis according to languages: (A) reintubation, (B) mortality, (C) ICU stay, (D)facial injury, (E) respiratory rate, (F) oxygenation index, (G) partial pressure of carbon dioxide. **Figure S2.** Subgroup analysis according to extubation method: (A) reintubation, (B) mortality, (C) ICU stay, (D)facial injury, (E) respiratory rate, (F) oxygenation index, (G) partial pressure of carbon dioxide. **Figure S3.** Subgroup analysis according to NIV parameter settings: (A) reintubation, (B) mortality, (C) ICU stay, (D) abdominal distension, (E) facial injury, (F) respiratory rate, (G) oxygenation index, (H) partial pressure of carbon dioxide. **Figure S4.** Subgroup analysis according to HFNC flow rate: (A) reintubation, (B) mortality, (C) ICU stay, (D) aspiration, (E) facial injury, (F) respiratory rate, (G) oxygenation index, (H) partial pressure of carbon dioxide.

## Data Availability

All data generated or analyzed in this study are included in this article.

## Abbreviations

HFNC: High-flow nasal cannula oxygen therapy
NIV: Non-invasive ventilation
LOS: ICU length of stay
PaCO_2_: Partial pressure of carbon dioxide
P/F: Oxygenation index
Rr: Respiratory rate
RCT: Randomized-controlled trial
RR: Risk ratio
95% CI: 95% confidence interval
MD: Mean difference
PRISMA: The preferred reporting items for systematic reviews and meta-analysis
SBT: Spontaneous breathing tests
APACHE II: Acute physiology and chronic health evaluation II
BMI: Body mass index
FD: Random‐effects model
RD: Random‐effects model
COPD: Chronic obstructive pulmonary disease
AECOPD: Acute exacerbation of chronic obstructive pulmonary disease
SAP: Severe acute pancreatitis
ARDS: Acute respiratory distress syndrome
IPAP: Inspiratory positive airway pressure
EPAP: Expiratory positive airway pressure
