# Supplementary material for: The efficacy of high-flow nasal cannula (HFNC) versus non-invasive ventilation (NIV) in patients at high risk of extubation failure: a systematic review and meta-analysis
Source: Eur J Med Res. 2023 Mar 14;28:120. doi: 10.1186/s40001-023-01076-9 (PMC10012596; doi:10.1186/s40001-023-01076-9)
Supplement: Supplementary file 2 — Additional file 2. Medline search. [file 40001_2023_1076_MOESM2_ESM.pdf]

Additional file 2 Medline search

#1	(((((("Noninvasive Ventilation"[Mesh]) OR ("noninvasive ventilation"[Title/Abstract])) OR ("non invasive ventilation"[Title/Abstract])) OR ("noninvasive positive pressure ventilation"[Title/Abstract])) OR ("non invasive positive pressure ventilation"[Title/Abstract])) OR ("niv"[Title/Abstract])) OR ("nippv"[Title/Abstract])
#2	((((((("humidified high flow nasal cannula therapy"[Title/Abstract]) OR ("high flow nasal cannulae"[Title/Abstract])) OR ("nasal high flow oxygen therapy"[Title/Abstract])) OR ("high flow nasal cannula oxygen therapy"[Title/Abstract])) OR ("high flow nasal cannula"[Title/Abstract])) OR ("hfnct"[Title/Abstract])) OR ("hfnc"[Title/Abstract])) OR ("hhfn"[Title/Abstract])
#3	#1 AND #2
