# Supplementary material for: The efficacy of high-flow nasal cannula (HFNC) versus non-invasive ventilation (NIV) in patients at high risk of extubation failure: a systematic review and meta-analysis
Source: Eur J Med Res. 2023 Mar 14;28:120. doi: 10.1186/s40001-023-01076-9 (PMC10012596; doi:10.1186/s40001-023-01076-9)
Supplement: Supplementary file 3 — Additional file 3: Figure S1. Subgroup analysis according to languages: (A) reintubation, (B) mortality, (C) ICU stay, (D)facial injury, (E) respiratory rate, (F) oxygenation index, (G) partial pressure of carbon dioxide. Figure S2. Subgroup analysis according to extubation method: (A) reintubation, (B) mortality, (C) ICU stay, (D)facial injury, (E) respiratory rate, (F) oxygenation index, (G) partial pressure of carbon dioxide. Figure S3. Subgroup analysis according to NIV parameter settings: (A) reintubation, (B) mortality, (C) ICU stay, (D) abdominal distension, (E) facial injury, (F) respiratory rate, (G) oxygenation index, (H) partial pressure of carbon dioxide. Figure S4. Subgroup analysis according to HFNC flow rate: (A) reintubation, (B) mortality, (C) ICU stay, (D) aspiration, (E) facial injury, (F) respiratory rate, (G) oxygenation index, (H) partial pressure of carbon dioxide. [file 40001_2023_1076_MOESM3_ESM.docx]

**Additional File 3. Subgroup analysis**

A


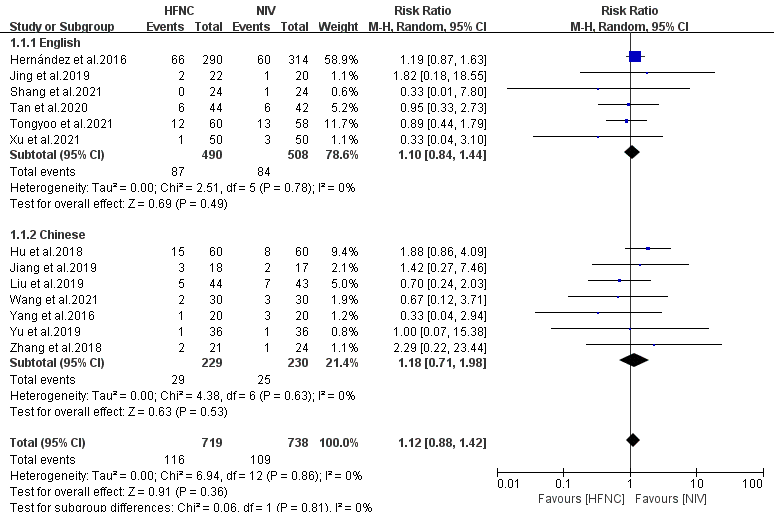


B


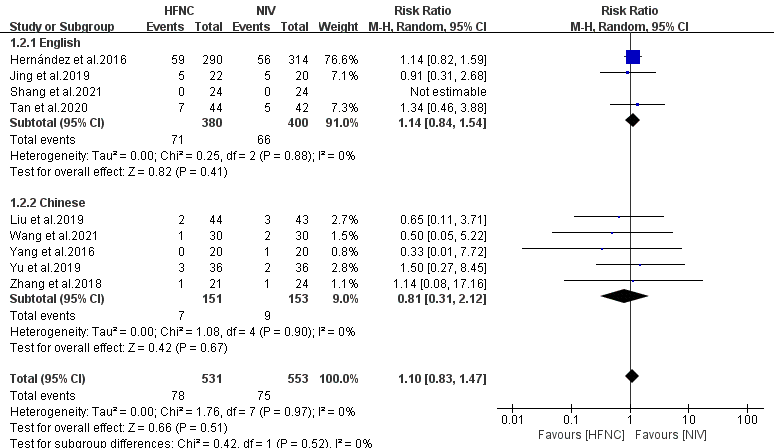


C


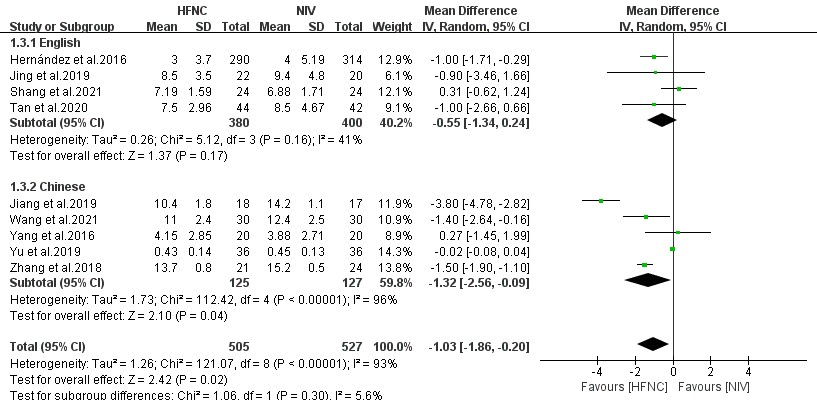


D


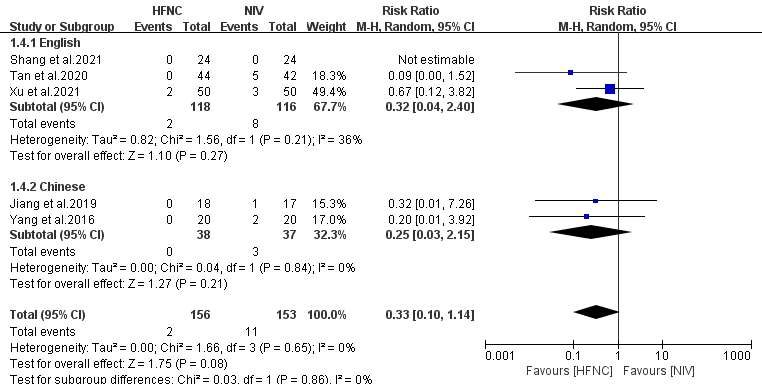


E


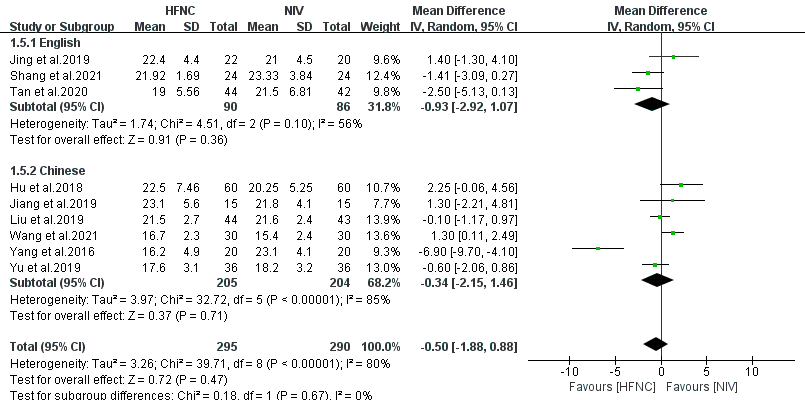


F
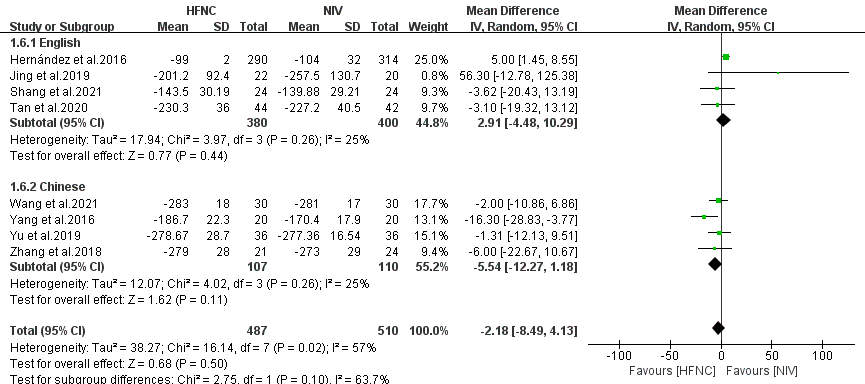


G
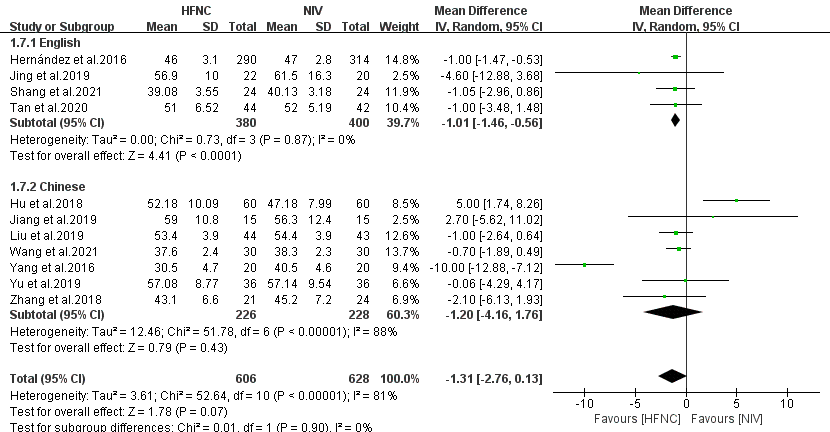


**Figure.1 Subgroup analysis according to languages: (A) reintubation, (B) mortality, (C) ICU stay, (D)facial injury, (E) respiratory rate, (F) oxygenation index, (G) partial pressure of carbon dioxide.**

A


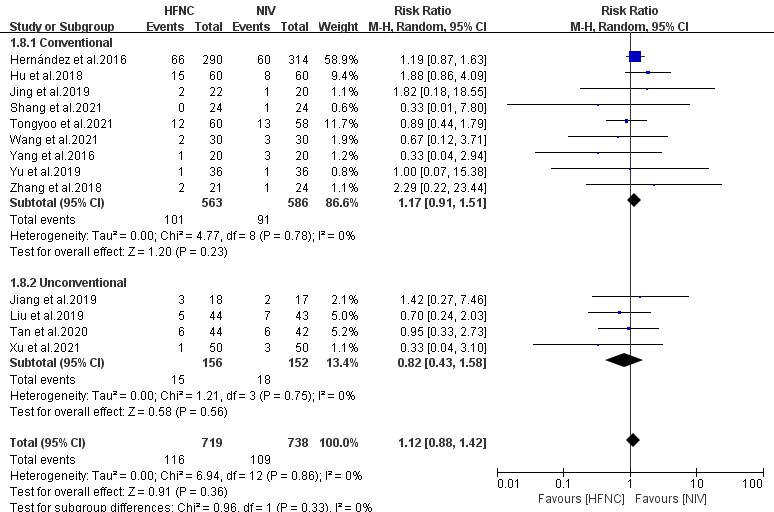


B


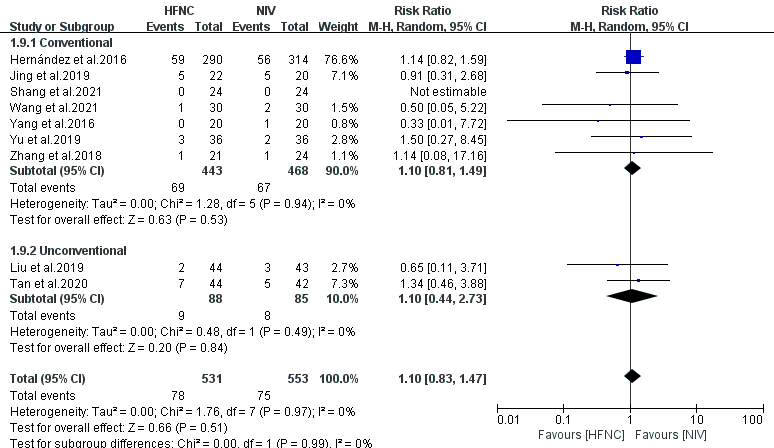


C


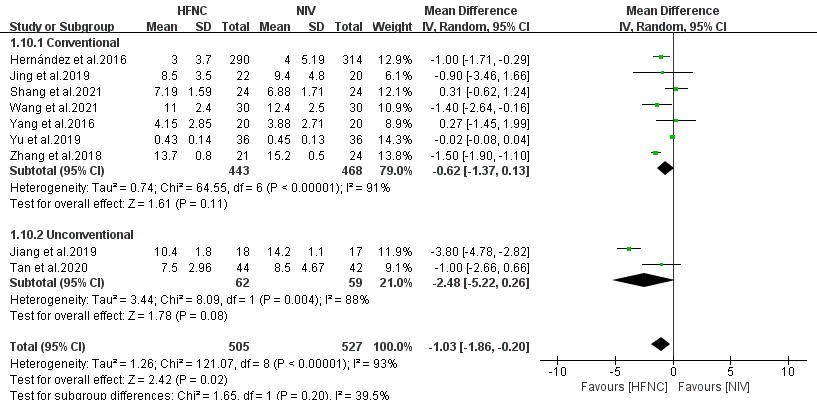


D


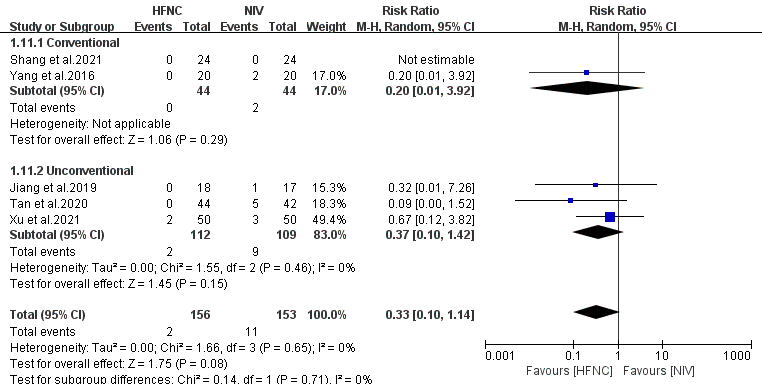


E


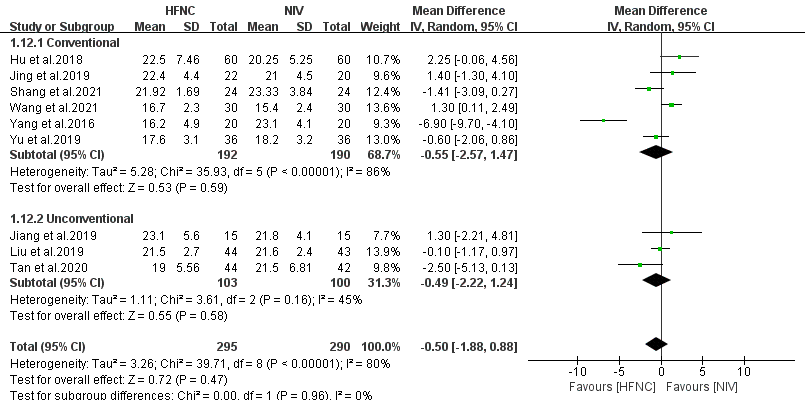


F


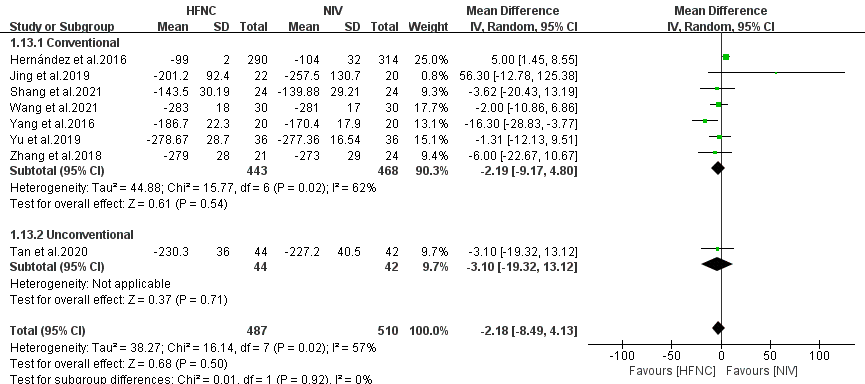


G


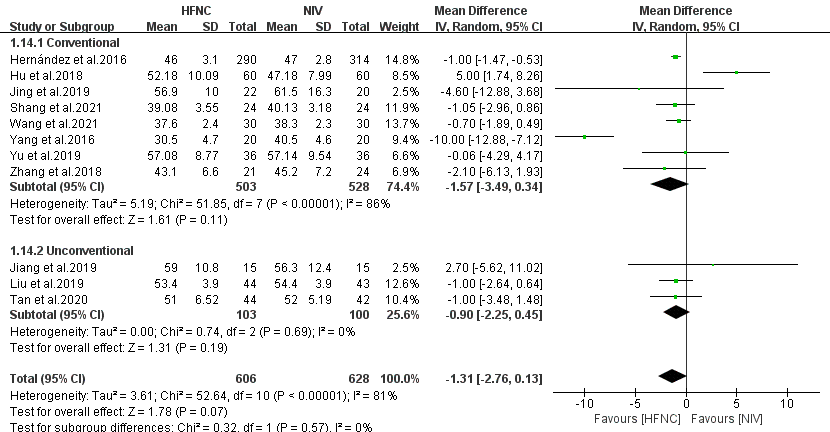


**Figure.2 Subgroup analysis according to extubation method: (A) reintubation, (B) mortality, (C) ICU stay, (D)facial injury, (E) respiratory rate, (F) oxygenation index, (G) partial pressure of carbon dioxide.**

A


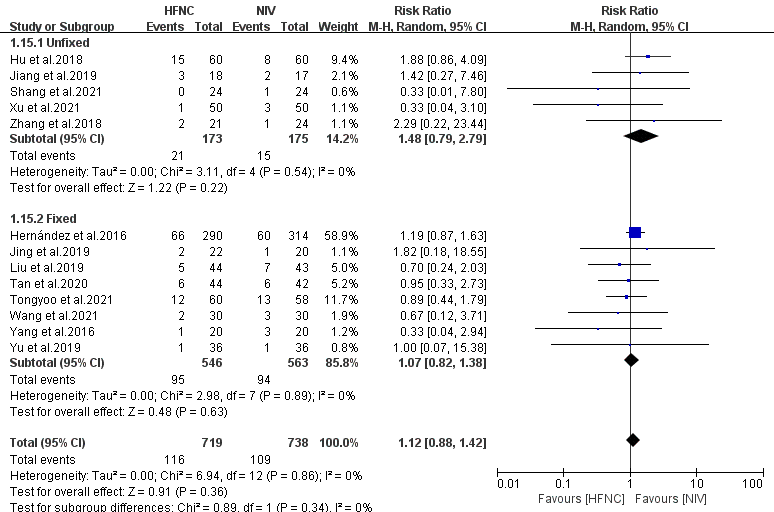


B


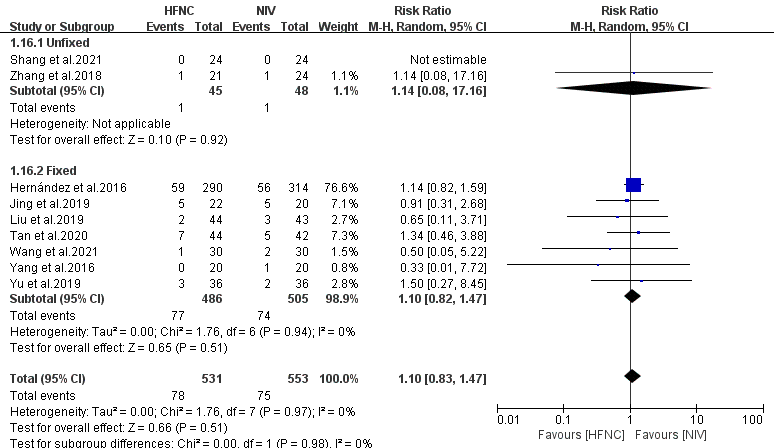


C


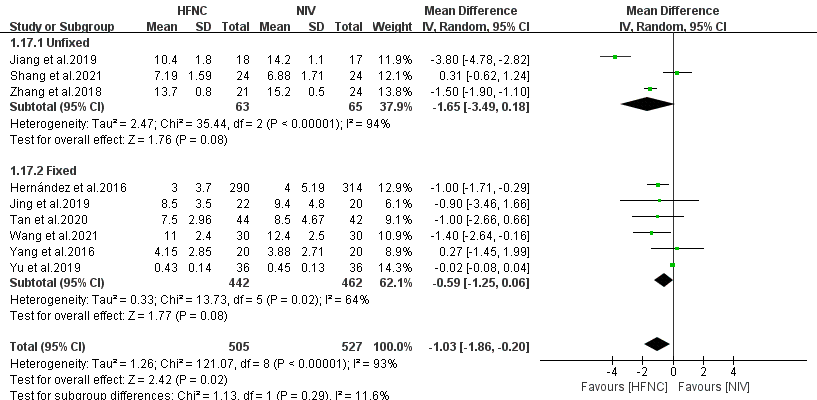


D


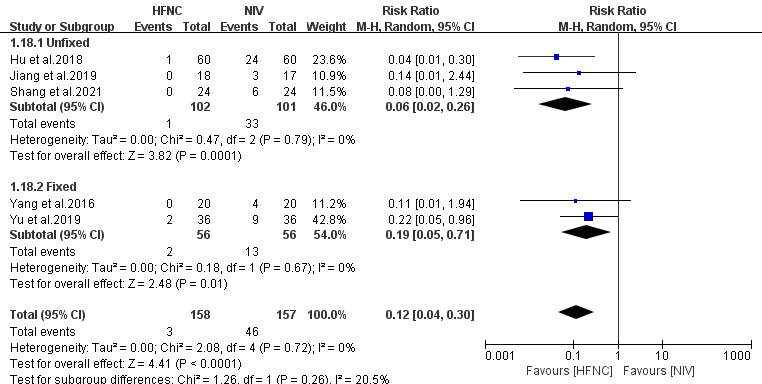


E


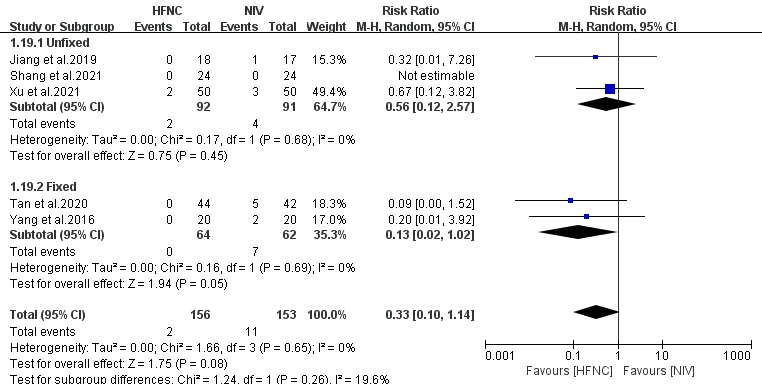


F


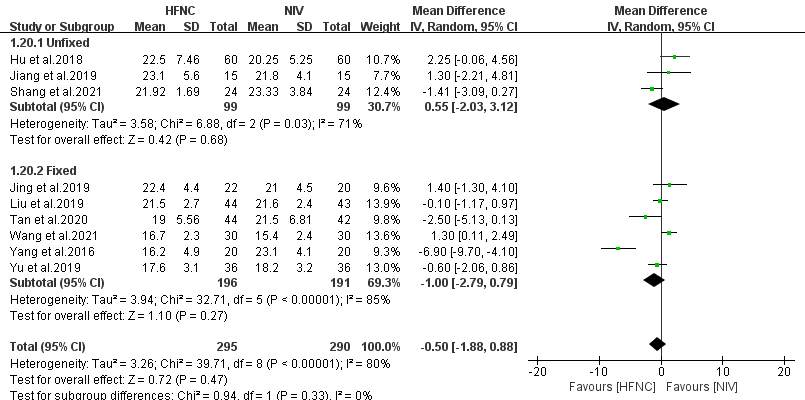


G


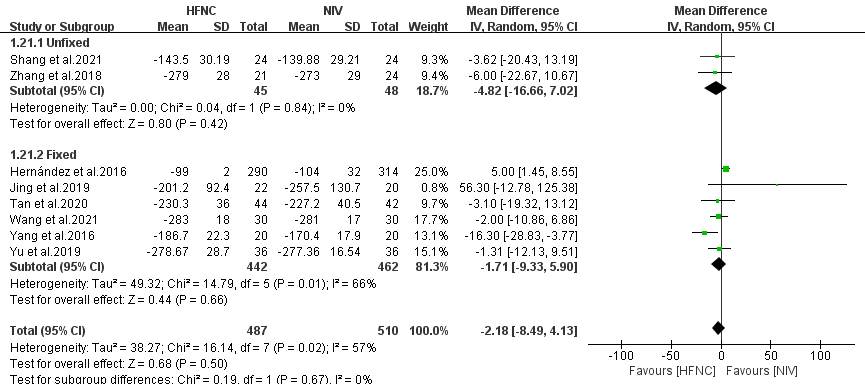


H


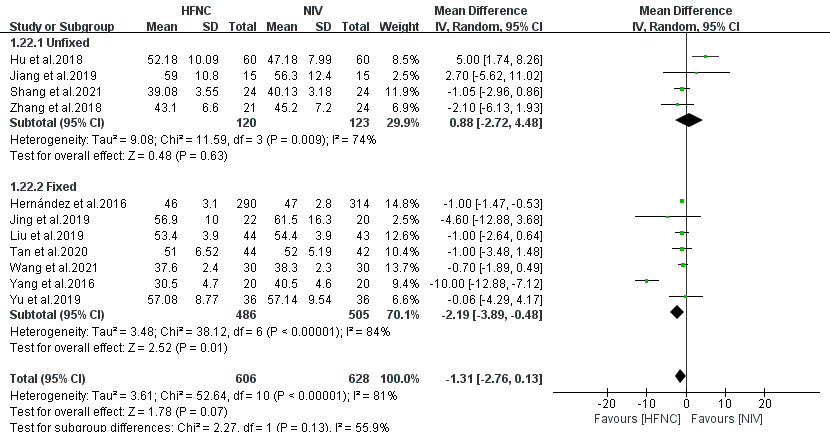


**Figure.3 Subgroup analysis according to NIV parameter settings: (A) reintubation, (B) mortality, (C) ICU stay, (D) abdominal distension, (E) facial injury, (F) respiratory rate, (G) oxygenation index, (H) partial pressure of carbon dioxide.**

A


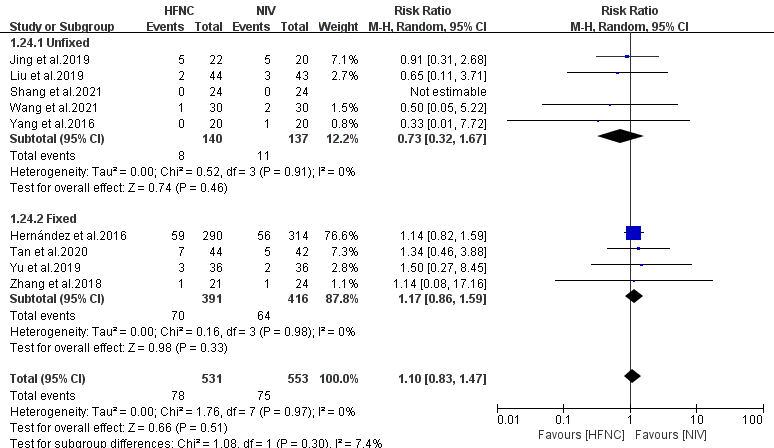


B


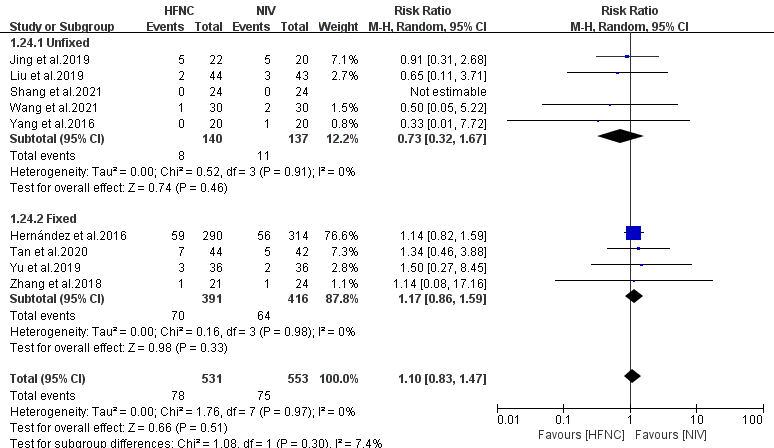


C


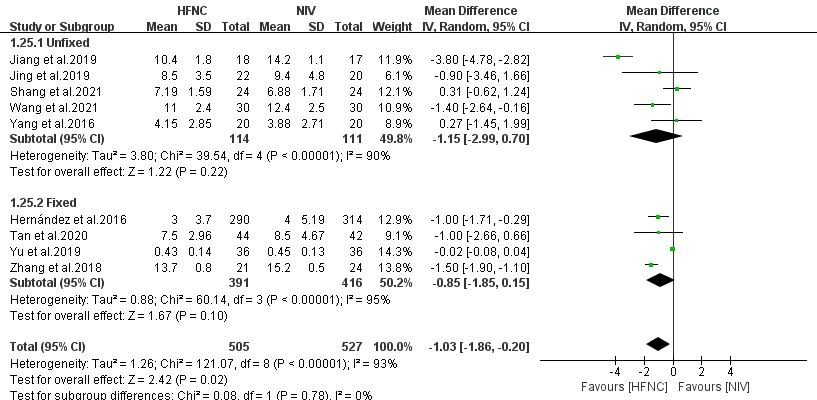


D


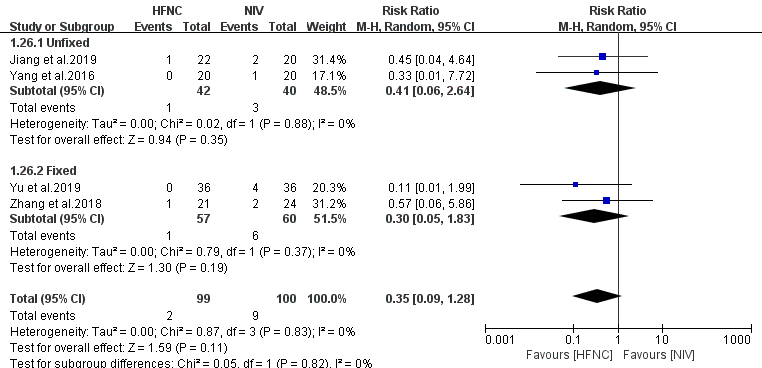


E


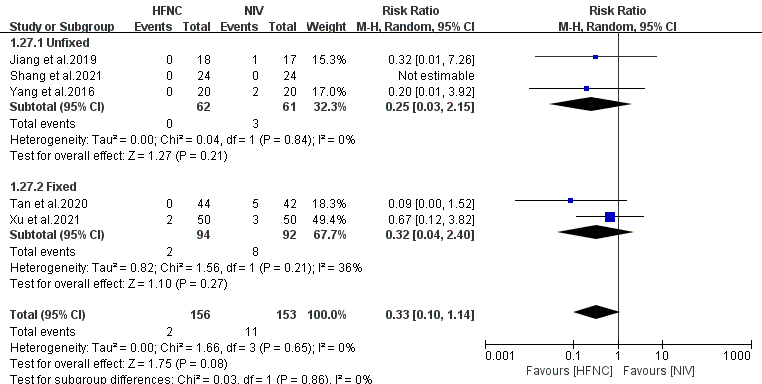


F


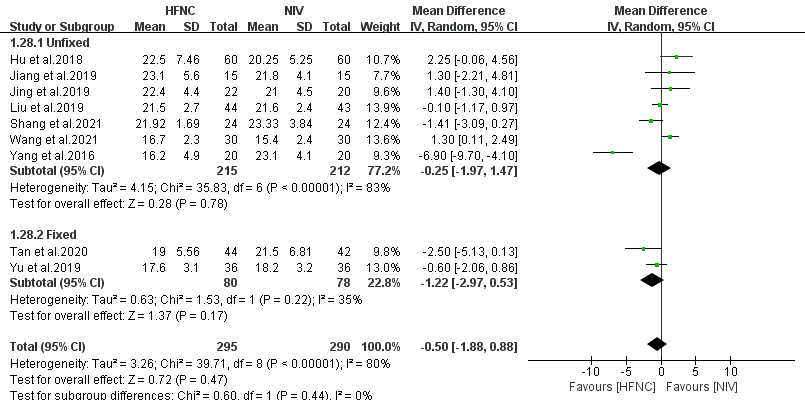


G


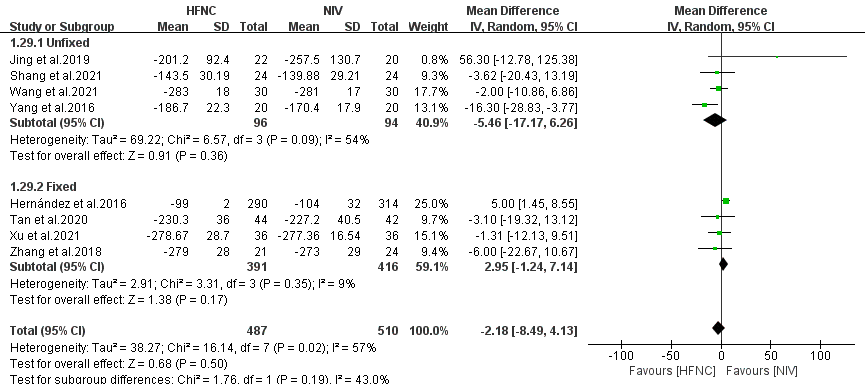


H


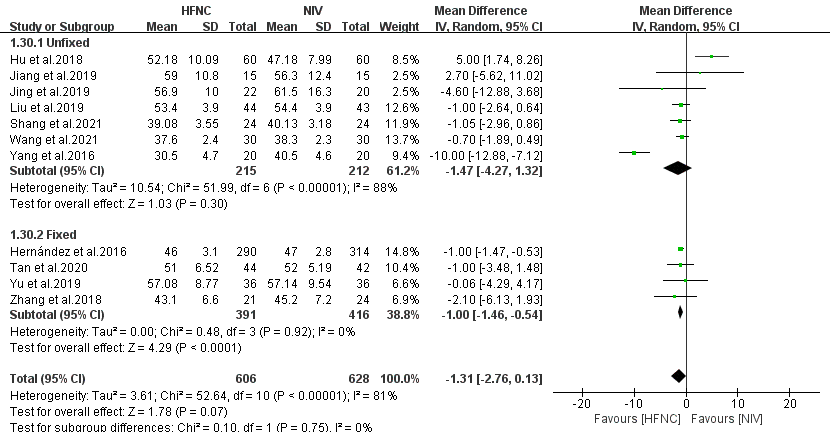


**Figure.4 Subgroup analysis according to HFNC flow rate: (A) reintubation, (B) mortality, (C) ICU stay, (D) aspiration, (E) facial injury, (F) respiratory rate, (G) oxygenation index, (H) partial pressure of carbon dioxide.**
